# Explaining face-voice matching decisions: The contribution of mouth movements, stimulus effects and response biases

**DOI:** 10.3758/s13414-021-02290-5

**Published:** 2021-04-01

**Authors:** Nadine Lavan, Harriet Smith, Li Jiang, Carolyn McGettigan

**Affiliations:** 1 Department of Biological and Experimental Psychology, School of Biological and Chemical Sciences, Queen Mary University of London, Mile End Road, London, E1 4NS UK; 2 Department of Speech, Hearing and Phonetic Sciences, University College London, 2 Wakefield Street, London, WC1N 1PF UK; 3 Department of Psychology, Nottingham Trent University, 50 Shakespeare Street, Nottingham, NG1 4FQ UK

**Keywords:** Face-voice matching, Cross-modal, Identity perception, Mouth movements

## Abstract

Previous studies have shown that face-voice matching accuracy is more consistently above chance for dynamic (i.e. speaking) faces than for static faces. This suggests that dynamic information can play an important role in informing matching decisions. We initially asked whether this advantage for dynamic stimuli is due to shared information across modalities that is encoded in articulatory mouth movements. Participants completed a sequential face-voice matching task with (1) static images of faces, (2) dynamic videos of faces, (3) dynamic videos where only the mouth was visible, and (4) dynamic videos where the mouth was occluded, in a well-controlled stimulus set. Surprisingly, after accounting for random variation in the data due to design choices, accuracy for all four conditions was at chance. Crucially, however, exploratory analyses revealed that participants were not responding randomly, with different patterns of response biases being apparent for different conditions. Our findings suggest that face-voice identity matching may not be possible with above-chance accuracy but that analyses of response biases can shed light upon how people *attempt* face-voice matching. We discuss these findings with reference to the differential functional roles for faces and voices recently proposed for multimodal person perception.

## Introduction

Faces and voices provide a wealth of information about a person. If some of the information about a person provided by faces and voices is redundant across the auditory and visual modalities (Collins & Missing, 2003; Saxton et al., 2006; Smith et al., 2016a; Yehia et al., 2002), it follows that it should be possible to match a face to a voice, even when a person is unfamiliar. Recent models of person perception emphasise the parallel and integrated nature of auditory and visual pathways, which interact as faces and voices are both processed for information about identity, speech and emotion (Belin, 2017; Belin et al., 2004; Young et al., 2020). The investigation of face-voice identity matching can thus shed light on how information from faces and voices is combined during multimodal person perception.

Previous studies have shown that face-voice identity matching is consistently above chance with dynamic (i.e. speaking) facial stimuli, but that performance is less likely to be above chance using static faces: For studies contrasting face-voice matching accuracy for dynamic and static faces, some have found that only dynamic face-voice matching is above chance level (Kamachi, Hill, Lander, & Vatikiotis-Bateson, 2003; Lachs & Pisoni, 2004). Others have shown that face-voice matching using static faces is also above chance (Krauss et al., 2002; Mavica & Barenholtz, 2013; Stevenage et al., 2017), particularly when matching procedures have a low memory load (Smith et al., 2016b). Such studies have observed numerical (but not statistical) disadvantages for static faces when compared to matching accuracy for dynamic faces (Smith et al., 2016a, 2016b, Experiment 3; Huestegge, 2019). Thus, while static images might at times be sufficient for accurate identity matching, overall the ability is fragile, and information included in dynamic faces may be key for reliable above-chance face-voice identity matching.

Dynamic faces uniquely convey information about articulatory mouth movements, which may be a cross-modal cue to identity, providing perceivers with a crucial link between the auditory and visual modalities. Previous research has established that dynamic articulatory cues can be mapped from one modality to another: For example, when a point-light technique is used to isolate articulatory mouth movements, participants are indeed able to match the dynamic light displays to auditory presentations of the same utterances (Rosenblum, Smith, Nichols, Hale, & Lee, 2006). Going beyond utterance matching, face-voice identity matching studies have furthermore indicated that it is possible to match a dynamic face to a voice saying a different sentence (Huestegge, 2019; Kamachi et al., 2003; Lander et al., 2007; Smith et al., 2016a, 2016b). These studies may suggest that, independent of the specific words they are saying, visual information about *how* a person speaks may be sufficient to match faces to voices: For example, if a person sounds like they are speaking very clearly or sounds like they are mumbling, this should be reflected in their mouth movements. Lander et al. (2001) indeed suggest that individual faces have “characteristic motion signatures”, which provide additional identity cues that in turn support identity perception. The availability of idiosyncratic articulatory mouth movement cues might also explain the higher accuracy usually observed for dynamic face-voice identity matching.

In this study, we initially set out to examine whether mouth movements can explain the advantage observed for dynamic face-voice matching over static face-voice matching. For this purpose, we conducted a face-voice matching experiment using a same-different procedure (see Smith et al., 2016a), which has a low memory load, and supports static and dynamic face-voice matching to a greater extent than other procedures (Smith et al., 2016b). We had two initial hypotheses:

- Hypothesis 1: Dynamic information in faces leads to above-chance face-voice matching accuracy.

In an effort to replicate the findings of a dynamic face-voice matching advantage from the previous literature, we presented participants with voices paired with dynamic videos or static images of faces. We predicted above-chance accuracy for dynamic face-voice matching when the whole face is visible, which would indicate that shared information is available across modalities to support identity matching. Based on the previous literature, it was unclear whether this advantage would also hold for matching between voices and static faces.

- Hypothesis 2: Mouth movements are essential to the advantage of dynamic face-voice matching, although other parts of the face still include relevant information.

Building on Hypothesis 1, we included two additional dynamic stimulus conditions (created from the video used in the dynamic whole-face condition) to examine the specific role of mouth movements in face-voice identity matching. In one condition, the articulating mouth was occluded – this allowed us to test whether identity matching is mediated by the perception and integration of speech articulations across modalities, as has been shown for speech comprehension (e.g. McGettigan et al., 2012). In the other condition, only the articulating mouth was visible, with the remainder of the image masked – this allowed us to test whether some part of the dynamic advantage reported in previous studies may be due in part to non-speech cues, for example to attractiveness or masculinity/femininity (Collins & Missing, 2003; Saxton et al., 2006; Smith et al., 2016a). We predicted higher accuracy for dynamic stimuli including mouth movements compared to the stimuli that did not include mouth movements. At the same time, we predicted lower accuracy for videos only showing the mouth region compared to dynamic faces showing the whole face, as much information about the face is lost when only showing the mouth.

To test these hypotheses, we used a highly controlled stimulus set, removing peripheral visual cues (hair and clothes), such that participants had to rely solely on facial cues during the matching task. We furthermore tested participants using more trials than previous studies (here 112, vs. 16 in Smith et al., 2016a), and more stimulus identities in order to overcome potential sampling issues at the stimulus level (Stevenage et al., 2017). We combined this highly controlled stimulus set with statistical analyses using generalised linear mixed models (GLMMs) to account for the random variation in the data introduced by design choices, different stimuli and participants.

Surprisingly, as reported in detail in the sections below, accuracy for all four conditions was no different from chance after accounting for random variation in the data, although above-chance performance was apparent when not accounting for this variation. Similarly, there was no difference in accuracy between any of the conditions. Crucially, however, chance-level accuracy does not indicate that participants were responding randomly in our task. Having employed a same-different procedure rather than a two-alternative forced-choice procedure, we were able to explore how response bias operates in face-voice matching. Distinct profiles of response behaviour were apparent in our data – a significant interaction of trial type (same vs. different) by condition indicated that participants were showing systematic response biases that varied by condition. Such response biases have been reported in previous face-voice matching studies employing a same-different procedure (Smith et al., 2016a, 2016c; Stevenage et al., 2017), where it has been observed that participants have an overall tendency to accept face-voice pairings as belonging to the same identity. Here, we therefore asked a second research question, addressed in an additional set of exploratory analyses: Beyond mouth movements, how are participants’ face-voice matching responses for static and dynamic faces affected by experimental design choices?

The study was pre-registered on the Open Science Framework (https://osf.io/4g25r).

In the sections that follow, we describe the methodology for the study, presenting pre-registered and exploratory analyses of accuracy (correct/incorrect), and exploratory analyses of response biases (same identity/different identity). We conclude that existing reports of face-voice identity matching may reflect, at best, a fragile ability in humans. Matching performance appears to be vulnerable to stimulus effects and is underpinned by distinct patterns of responses dependent on the nature of the visual stimuli. These responses may consequently manifest as above-chance performance only when considering raw accuracy scores.

## Methods

### Participants

One hundred and nine participants were recruited via the online recruitment platform *Prolific.co*. All participants were aged between 18 and 40 years (mean age = 28.9 years, SD = 6.45; 56 female), were native speakers of English with no reported hearing difficulties, and had a high approval rate on Prolific (> 90%). Ethical approval was given by the local ethics committee (Project ID number: SHaPS-2019-CM-029). One participant was excluded as they missed more than 20% of the catch trials (see *Procedure*). Each participant was paid £3.40 for 27 min of participation. For this final sample of 108 participants, 27 participants were randomly assigned to each of the four conditions (Whole Face (static), Whole Face (dynamic), Mouth Only (dynamic), Mouth Occluded (dynamic)).

### Materials

The face and voice stimuli were sourced from the GRID audio-visual sentence corpus (Cooke, Barker, Cunningham, & Shao, 2006). This corpus contains high-quality audio-visual recordings of 1,000 sentences spoken by 34 talkers (18 male, 16 female; Cooke et al., 2006). Each sentence has the same structure: (1) command, (2) colour, (3) preposition, (4) letter, (5) digit, and (6) adverb, such as “put red at G9 now”. Audiovisual and audio clips are available in this corpus. To avoid any confounding effect of ethnicity, we excluded two Non-White male identities from the experiment. A further two male identities were used in practice trials, leaving 14 White male identities for use in the main experiment. For the experimental stimulus set, we randomly selected four videos and four audio clips from each of these 14 White male speakers, as well as 14 White female speakers from the corpus – all items were unique, i.e. none of the sentences used was repeated within the experiment, either within or across modalities. Audio tracks were converted to MP3.

**Fig. 1:**
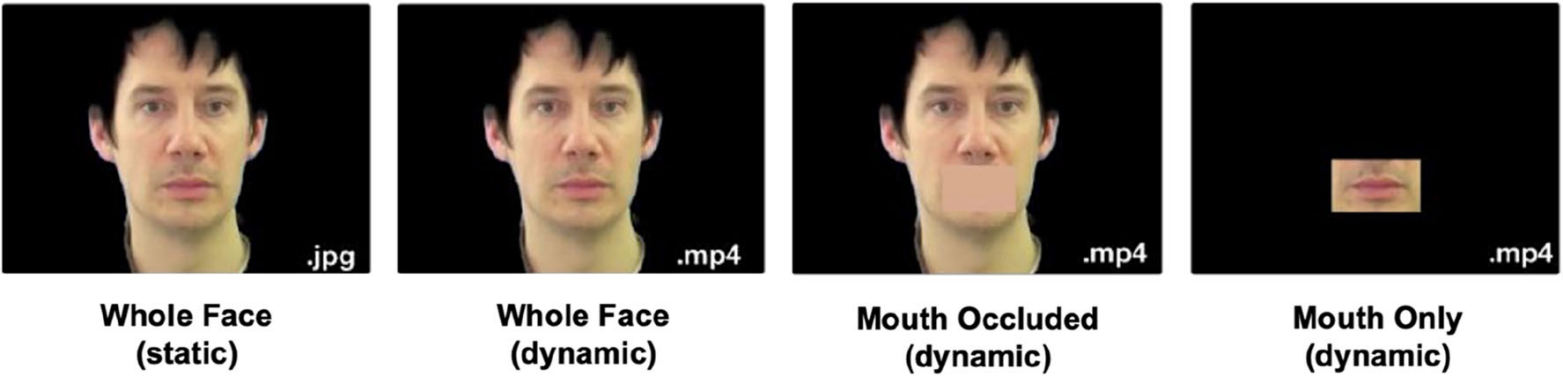
Illustration of the four visual conditions included in the study

To create the face stimuli, we first pre-processed the audio-visual stimuli. In the original audio-visual recordings, there was some variability in the position of recorded individuals in relation to the camera. We therefore first centred the faces in all videos and scaled the size of the faces to be similar across the individuals portrayed. From these centred and scaled videos, we then created muted videos for our four visual conditions. Examples of the stimuli per condition are shown in Fig. 1.

For the Whole Face (dynamic) condition, we used Adobe Premiere CC 2018 (version 12.0) to remove the hair, clothing, and background information from the videos, thus including only information about the face in our stimuli. From the Whole Face (dynamic) stimuli, we extracted individual frames to create the Whole Face (static) stimuli. These frames were selected to include a relatively neutral facial expression, avoiding speech-related movements. For the Mouth Occluded (dynamic) stimuli, we masked the mouth of each speaker with a rectangle of their average skin colour. The size of mask was manually adjusted to ensure that the mouth was fully covered for all individuals. For this condition, all information about the dynamic mouth movements was therefore excluded. Finally, we created stimuli for the Mouth Only (dynamic) condition, in which we only included the information from the small rectangular area including the mouth (i.e. the opposite of the Mouth Occluded condition), with the rest of the video blacked out. This condition therefore included only information about the mouth movements. All videos were muted and exported at a 720 pixel x 576 pixel resolution (4:3 ratio) with a sample rate of 25 frames/s. All videos were 3 s in length (Cooke et al., 2006). In the task, static images were shown for 2 s as a viewing time of 3 s made the task appear slow-moving during pilot testing.

### Procedure

**Fig. 2:**
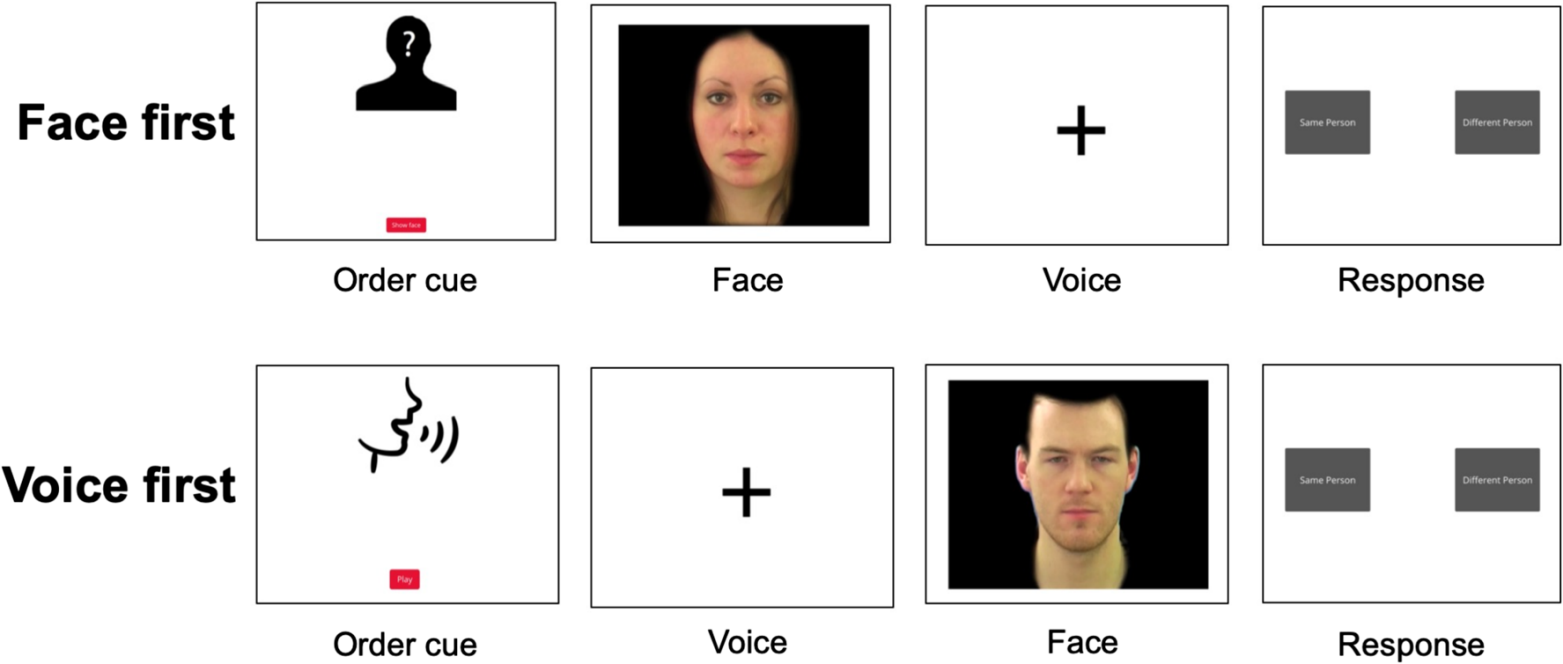
Overview of the trial structure for the experiment: The upper and lower rows illustrate trials in which the order of stimuli was face-then-voice (‘face first’) and voice-then-face (‘voice first’), respectively

The task was completed in the Gorilla Online Experiment Builder (gorilla.sc, Anwyl-Irvine et al., 2018). After giving informed consent, participants completed headphones screening (Woods et al., 2017). Condition was manipulated between subjects, such that participants were randomly assigned to complete a face-voice matching task including one of the four visual conditions. For the face-voice matching tasks, participants were presented with a pair of stimuli, including one voice recording and one muted dynamic video or static image, one after the other. Half of the pairs featured the same identity, the other half featured two different identities. The order of modalities was counterbalanced and participants were cued as to whether the current trial would start with a voice recording or a muted video. After the stimulus presentation, participants were then prompted to judge whether the two stimuli showed the same person or two different people via a mouse click on response buttons labelled ‘same person’ and ‘different person’, respectively (see Fig. 2). Before completing the main task, participants completed three practice trials to familiarise themselves with the task. In the main task, we furthermore included 12 vigilance trials (see exclusion criteria) at random intervals. In these vigilance trials, participants were either asked via text appearing on the screen to “please follow the instruction in the audio channel” or via a voice recording to “please follow the instruction in the following image”. They were asked to either respond by clicking the ‘same person’ or ‘different person’ button. In this way, we could ensure that participants would attend to both modalities in every trial. In total there were 124 trials, including the 12 vigilance trials. To counterbalance identities across the different pairs of identity, we made four versions of the task, each including different identity pairs for the different-identity trials (e.g. one participant would encounter ID1 paired with either ID2 or ID3 in the different-identity trials, while another might encounter ID1 paired with either ID4 or ID5). Although pairings were not exhaustive, these pairings were created in this systematic way to ensure that as many identity pairs as possible were sampled in our study (across participants). The four versions were counterbalanced across participants. We furthermore ensured that the same identity was not presented in consecutive trials. After the main task, participants were asked to complete a brief questionnaire about their experience of the experiment. The data from this questionnaire were part of a student research project and are not analysed for the purpose of this paper.

## Results

### Research Question 1: Do mouth movements contribute to more accurate identity matching for faces and voices?

- Hypothesis 1: Dynamic information in faces leads to above-chance face-voice matching accuracy.

Based on the previous literature (Kamachi et al., 2003; Lachs & Pisoni, 2004; Lander et al., 2007), we predicted that accuracy for face-voice matching with dynamic stimuli showing the whole face would be above chance, while we had no specific predictions as to whether we would also find above-chance performance for matching of static faces or the remaining dynamic conditions.

Mean accuracy, averages across ‘same identity’ and ‘different identity’ was low across all conditions (52–57% accurate), which appears to be broadly in line with other reports in the literature.

**Fig. 3:**
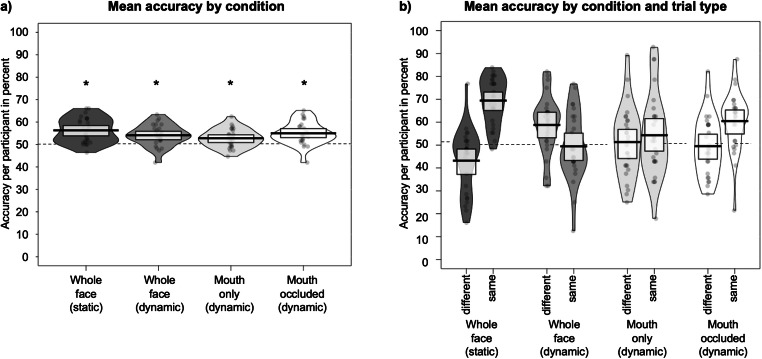
**a** Mean accuracy (%) per participant for the four conditions. **b** Mean accuracy (%) per participant for the four conditions plotted by trial type (same/different). Chance performance is at 50% (dashed line). Boxes show 95% confidence intervals. * indicates p < .05 for the one-sample t-tests comparing accuracy against chance. Note that the accuracy is, however, not above chance in our exploratory analysis using generalised linear mixed models

In a confirmatory analysis, we entered each participant’s overall mean accuracy into one-sample t-tests against chance (50% correct) for each of the four conditions (Whole face (static), Whole face (dynamic), Mouth only (dynamic), Mouth occluded (dynamic)). These t-tests showed that for all conditions, accuracy was significantly above chance (all *t*s(26) > 3.5, all *p*s < .003). Means per condition are plotted in Fig. 3a. Crucially, however, a shortcoming of this statistical analysis is that one-sample t-tests cannot simultaneously account for stimulus and participant effects (see Smith et al., 2016a; Wells et al., 2013), even though participants are likely to vary in their ability to match faces and voices, and some stimuli or identities are likely to be easier to match to one another than others. Random variance due to one or more of these factors may therefore affect the results of the t-tests.

To account for this kind of random variation, we ran a GLMM using the *lme4* package (Bates et al., 2015) in the *R* environment to further assess whether accuracy for these conditions is truly above chance, even after accounting for such random effects. In this GLMM, we entered condition as a fixed effect. Participant, trial type (same or different identity), the voice stimulus nested within identity, as well as the face stimulus nested within identity were entered as random effects. Other random effects, such as presentation order (face-first/voice-first), were not included as they led to singular fits or issues with model convergence.

We obtained 95% confidence intervals (CIs) by simulating the posterior distributions of the cell means in *R* (*arm* package, version 1.6; Gelman & Su, 2013) to assess whether accuracy was above chance. Confidence intervals for all conditions included chance performance (50%; all CIs [<.50; >.59]), indicating that the accuracy for all of the conditions is in fact *not* significantly different from chance.

The above-chance performance found in the one-sample t-tests seems to therefore be largely driven by the stimulus effects accounted for by the random-effects structure of the GLMM. These results are therefore not in line with our prediction that videos of dynamic faces would result in above-chance accuracy in this face-voice matching task.

- Hypothesis 2: Mouth movements are essential to account for an advantage of dynamic face-voice matching, although other parts of the face still include relevant information.

We also hypothesised that the information encoded in mouth movements drives differences in accuracy between the different conditions. We therefore predicted lower accuracy for dynamic stimuli with no information about mouth movements compared to dynamic stimuli including mouth movements. Given that much information about a face is lost in stimuli only showing the mouth, we additionally predicted that accuracy would be lower for videos only showing the mouth region compared to dynamic faces showing the entire face. Mean accuracy per condition and trial type are plotted in Fig. 3b.

To address our predictions in a confirmatory analysis, we ran another GLMM to contrast accuracy for the different conditions with each other – split by trial type. Trial-wise accuracy was the outcome variable, condition was entered as a fixed factor. We now also included trial type (same or different identity) and an interaction of trial type and condition fixed effects based on previous studies showing differences in accuracy varying along these two factors (Smith et al., 2016a, 2016c; Stevenage et al., 2017). The random-effects structure was the same as described above for the exploratory analysis, with only trial type having been moved into the fixed effects as it now became an effect of interest. This random-effects structure differs from the preregistered random-effects structure due to issues with singular fits and model convergence. Significance of the main effects and interactions was established via log-likelihood tests by dropping effects of interest from the appropriate model. For example, to test for the significance of the two-way interactions we dropped the interaction term from a model that included all main effects.

The model output is shown in Table 1. We found a significant interaction between condition and trial type (χ^2^[3] = 192.01, p < .001). In the presence of an interaction, main effects are of limited interpretability and were therefore not tested. A visual inspection of Figs. 3a and b, however, shows that although trial type and condition interact, neither are there clear condition-wise advantages for dynamic relative to static faces nor did occluding the mouth have an obviously detrimental effect on accuracy. From these analyses, we can therefore conclude that there is no evidence in our data that face-voice matching is driven or influenced by shared information encoded in mouth movements. This lack of a difference in accuracy by condition is likely linked to our previous finding that overall accuracy was not significantly different from chance for any of the conditions.

**Table 1:** Coefficients and standard errors (reported on a log-odds scale) for the generalised linear mixed model (GLMM) for the analysis of the effects of condition and trial type on accuracy^a^

Predictors	Log-Odds	Standard Error
(Intercept)	-0.28	0.09
Main effect of Trial Type		
Trial Type (Same)	1.13	0.08
Main effect of Condition		
Condition (Whole Face (Dynamic))	0.65	0.11
Condition (Mouth Only (Dynamic))	0.34	0.11
Condition (Mouth Occluded (Dynamic))	0.26	0.1
Interaction of Trial Type and Condition		
Trial Type (Same) * Condition (Whole Face (Dynamic))	-1.51	0.11
Trial Type (Same) * Condition (Mouth Only (Dynamic))	-1.01	0.11
Trial Type (Same) * Condition (Mouth Occluded (Dynamic))	-0.68	0.11

^a^The reference category for Trial Type is the ‘different’ trials. The reference category for Condition is Whole Mouth (Static)

**Fig. 4:**
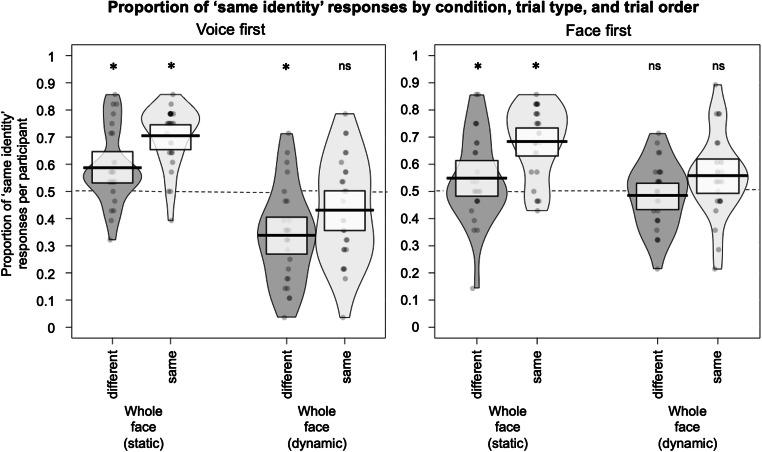
Proportion of ‘same identity’ responses per participant for the dynamic and dynamic Whole Face conditions, split by trial type (same/different). The left-hand plot shows the data for trials where the voice was presented first, the right-hand plot shows the data for trials where the face was presented first. Boxes show 95% confidence intervals. Asterisks indicate that the proportion of ‘same identity’ responses is different from 0.5

Although accuracy on all four conditions was no different from chance, the two-way interaction indicates that substantial biases in participant responses are apparent across trial types for some of the conditions (see Fig. 4). The existing literature on face-voice matching has reported on the presence of similar response biases in relation to trial types and stimulus order (Smith et al., 2016a, 2016b, 2016c; Stevenage et al., 2017). In a set of exploratory analyses, we therefore set out to formally examine how participants’ responses are affected by different aspects (trial type, stimulus order) of our experimental design.

### Research Question 2: How are participants’ responses to dynamic and static faces affected by trial type and stimulus order?

For the following exploratory analyses, we dropped the Mouth Only (dynamic) and Mouth Occluded (dynamic) conditions from our analyses, focusing on the static and dynamic Whole Face conditions. We did this as the Mouth Occluded and Mouth Only conditions were originally included to explore the effects of mouth movements on accuracy in face-voice matching, a question that was no longer relevant for these exploratory analyses. Furthermore, while accuracy was our measure of interest in the previous set of analyses, we now analysed the raw same/different responses per participant to facilitate analyses of biases. The proportion of ‘same identity’ responses is plotted by trial type (same/different), stimulus order (voice first/face first), and condition (static image/dynamic video) in Fig. 3b.

### Exploratory analysis 1: How does participants' response behaviour relate to chance performance for different trial types and orders?

We assessed the response biases by comparing the proportion of ‘same’ responses to 0.5 (equal proportion of ‘same identity’ and ‘different identity’ responses). For this purpose, we ran a no-intercept GLMM to examine the effects of stimulus order, trial type and condition on participants’ responses (raw ‘same’ or ‘different’ responses). We modelled all interactions and included the same random effects structure as described above. We then again obtained 95% CIs by simulating the posterior distributions of the cell means in *R*. All CIs including 0.5 indicate that participants gave a similar proportion of ‘same identity’ and ‘different identity’ responses for the relevant condition.

For static images of faces, participants are overall biased to perceive face-voice pairs as the same identity (see Fig. 4). As would be expected for accurate matching, participants gave more ‘same identity’ responses for ‘same identity’ trials, for both stimulus orders (voice first: CIs [0.66; 0.79]; face first: CIs [0.64; 0.77]). However, for ‘different identity’ trials there was no corresponding preference to give ‘different identity’ responses: For trials in which the face was presented first, ‘same identity’ and ‘different identity’ responses were equivocal, with the CI including 0.5 (CIs [0.48; 0.63]). Intriguingly, for ‘different identity’ trials in which the voice was presented first, participants gave a higher proportion of ‘same identity’ response (CIs [0.52; 0.66]).

For dynamic videos of faces a different pattern of biases emerges: Participants more frequently perceive face-voice pairs as *different* identities compared to responses for static faces, an effect that is particularly pronounced for trials where the voice was presented first. Specifically, for ‘same identity’ trials, participants’ responses were not significantly different from 0.5 for both stimulus orders (voice first: CIs [0.36; 0.50]; face first: CIs [0.49: 0.63]), indicating that ‘same identity’ and ‘different identity’ responses were equivocal. For ‘different identity’ trials, there were more ‘different identity’ responses when the voice was presented first (CIs [0.25; 0.38]), but when the face was presented first the ‘same identity’ and ‘different identity’ responses were equivocal (CIs [0.41; 0.56]). Response biases therefore appear to differ both by condition and stimulus order.

### Exploratory analysis 2: How do stimulus order, trial type, and condition affect response behaviour?

We ran another intercept-only GLMM with the same structure as above to explore how stimulus order, trial type and condition affect response behaviour.

Neither the three-way interaction (χ^2^[1] = 1.17, *p* = .280), nor the two-way interaction of trial type and condition (χ^2^[1] = 3.49, *p* = .067), nor the two-way interaction of trial type and order (χ^2^[1] = .305, *p* = .581) were significant. There was, however, a significant two-way interaction for condition and stimulus order (χ^2^[1] = 42.46, *p* < .001). This interaction can be explained by the findings from Exploratory Analysis 1 above: Dynamic face-voice pairs are more often perceived as different identities when the voice is presented first. In contrast, participants’ bias to respond ‘same identity’ for static faces is largely independent of the stimulus order

Since trial type did not interact with any of the remaining factors, we also tested for this main effect. This showed that, despite the overall chance-level performance, participants indeed gave more ‘same identity’ responses for trials that included the same identity than for ‘different identity’ trials (χ^2^ = 73.33, p < .001). For the full model outputs, please see Table 2.

**Table 2:** Coefficients and standard errors (reported on a log-odds scale) for the full generalised linear mixed model including the three-way interaction for the analysis of the effects of condition, trial type and order on response behaviour^a^

Predictors	Log-Odds	Standard Error
(Intercept)	0.4	0.16
Main effect of Trial Type		
Trial Type (Same)	0.58	0.12
Main effect of Condition		
Condition (Whole Face (Dynamic))	-1.18	0.22
Main effect of Order		
Order (Face First)	-0.17	0.12
Interaction of Trial Type and Condition		
Trial Type (Same) * Condition (Whole Face (Dynamic))	-0.08	0.17
Interaction of Trial Type and Order		
Trial Type (Same) * Order (Face First)	0.06	0.17
Interaction of Condition and Order		
Condition (Whole Face (Dynamic)) * Order (Face First)	0.89	0.17
Interaction of Trial Type, Condition, and Order		
Trial Type (Same) * Condition (Whole Face (Dynamic)) * Order (Face First)	-0.26	0.24

^a^The reference category is ‘different identity’ trials for Trial Type, Whole Mouth (Static) for Condition, and Voice First for Order

## Discussion

In this study, we used a same-different face-voice matching paradigm to address two main hypotheses. First, we aimed to test whether accuracy for face-voice matching would be above chance for dynamic faces in a same-different task. Second, we tested the proposal that dynamic face-voice matching might be explained, partly or in full, by the perception and integration of articulatory cues across the two modalities. However, the matching accuracy analysis revealed no evidence for above-chance performance in any of the conditions, nor did we find any differences in accuracy between conditions. We therefore do not replicate the frequently reported above-chance accuracy for dynamic face-voice matching (Kamachi et al., 2003; Lachs & Pisoni, 2004; Smith et al., 2016a, 2016b) and were therefore not able to directly explore whether mouth movements can explain such an advantage. Nevertheless, our overall results provide important insights into the cognitive processes underpinning face-voice matching decisions.

In follow-up exploratory analyses of response biases – specifically, the probability of participants responding ‘same’ to face-voice pairings across condition, trial type and stimulus order – we found evidence for differential response biases when face stimuli were dynamic versus static. These response biases indicate that participants were by no means making random matching decisions, as may be concluded from the chance-level accuracy. Participants’ responses were systematically affected by aspects of the experimental design. Overall, our findings therefore suggest that humans struggle to accurately map identity representations between unfamiliar face and voice stimuli, but that their decision-making is affected systematically by task and stimulus properties.

### Face-voice matching accuracy

Our finding that participants cannot match identity across (dynamic or static) faces and voices with above-chance accuracy partially conflicts with the extant literature. We note, however, that the current study differs from previous work in several key ways, which may explain these discrepant results. We implemented a number of design and analysis choices to support and focus in on the detection of face-voice matching ability within a tightly controlled experiment. To this end: (1) We used a larger number of trials and identities than some of the previous studies to test the generalisability of matching performance; (2) we minimised identity cues extraneous to the face by masking out of hair and other non-facial features, as well as standardising the size and position of images onscreen; and (3) we modelled aspects of the design as random effects in our statistical analyses (see also Smith et al., 2016a; Wells et al., 2013). Crucially, simple t-tests indicated that face-voice matching accuracy in our study was above chance level. However, when modelling random effects of participant, identity, and stimulus to avoid Type 1 error inflation (Baguley, 2012; Clark, 1973; Judd et al., 2012), confidence intervals for accuracy crossed chance-level performance in all conditions. Previous work has shown that accuracy on face-voice matching varies substantially depending on the talker identity or specific stimuli (Mavica & Barenholz, 2013; Smith et al., 2016a, 2016b, 2016c; Stevenage et al., 2017). Taking this observation and our findings together, we suggest that some studies reporting above-chance accuracy may indeed be strongly influenced by stimulus effects (although we do not rule out that other design and stimulus choices may affect accuracy). Thus, while there may be diagnostic cues to identity that are perceptible across modalities for some talkers, this does not appear to be the case for all identities. While previous studies have accounted for such stimulus variability in their statistical analyses and have observed above-chance face-voice matching accuracy (Smith et al., 2016a, 2016b), issues relating to stimulus variability are still likely to account for the discrepancy with our set of results: We used a larger set of stimuli and implemented substantially more trials than Smith et al. (2016a, 2016b).

The effect of stimulus variability is unsurprising: Studies that have attempted to pinpoint salient visual and auditory identity cues have reported that the weight of these cues might vary across perceivers and listening/viewing situations (Mathias & von Kriegstein, 2014; Kreiman & Sidtis, 2011; Burton et al., 2016). For example, while cues to masculinity may be correlated across the face and voice (Smith et al., 2016a), these might only support face-voice matching in more extreme cases (e.g. a voice with very low pitch is likely to match a face with a pronounced brow ridge). In contrast, identities closer to the norm in terms of masculinity might display less redundant information across modalities, such that there are fewer sexually dimorphic cues available to predict how the acoustic patterns in that person’s speech might map onto a view of their face. Whether or not visual cues to masculinity were accessible from the identities in our study, removing information about the hair and clothes may have removed additional cues and could thus have contributed to lowering accuracy to chance level, even for the dynamic videos showing the whole face. This may further explain why our results appear inconsistent with previous studies. While Mavica and Barenholtz (2013) observed above-chance static face-voice matching with hair and clothing cues removed, their analyses did not include stimulus as a random effect. Descriptively speaking, accuracy was higher when hair and clothing were included.

### Response biases in face-voice matching

Beyond our research question of the contribution of mouth movements, we found an interactive effect of face condition (static vs. dynamic) and stimulus order (i.e. face first vs. voice first) on task responses, as revealed by a set of exploratory analyses. We found that the responses were biased towards responding ‘same’ for static trials, irrespective of the stimulus order. Additionally, we found responses to be equivocal or biased toward ‘different’ responses for dynamic stimuli. These results are broadly aligned with recent studies that have used a same-different procedure (Smith et al., 2016a, 2016c; Stevenage et al., 2017) to explore response biases in face-voice matching. The results of Smith et al. (2016a) point to an overall bias to respond ‘same’ in sequential same-different tasks, with participants reported to be more accurate at detecting a ‘match’ than a ‘mismatch’ for both dynamic and static faces. Similarly, Stevenage et al. (2017) applied a signal-detection analysis to simultaneous same-different judgements from a static face-voice matching task, also revealing a significant bias to respond ‘same’. Furthermore, previous same-different face-voice matching tasks report effects of stimulus order: Accuracy has been reported to differ according to order, with results suggesting that the bias to respond ‘same’ is most pronounced when the face is presented before the voice (Smith et al., 2016a, 2016c).

We speculate that these response biases can illuminate how information from faces and voices interact during identity perception. While face and voice perception might be integrated processes, they are not identical (Stevenage & Neil, 2014): Voice perception contributes more to speech analysis, and face perception arguably contributes more to identity analysis (see Young et al., 2020). On this basis, how may our observed interaction between condition and order relate to the varying functional role of faces and voices in everyday life, as described by Young et al. (2020)? Identity perception accuracy is higher for faces than voices (Hanley & Damjanovic, 2009; Stevenage et al., 2011). This has been proposed to be because of differential link strength in the face and voice perception pathways (Damjanovic & Hanley, 2007; Stevenage et al., 2012), and because mental representations of voice identity are weakly encoded in comparison to face identity (Stevenage et al., 2011; Stevenage et al., 2013). In a same-different matching task, if we rely on the face to indicate identity, then identity representations perceived from the accompanying voice might be ill-formed and non-specific. A voice might therefore be typically accepted as coming from the same identity as the face if identity information rather than speech information is being used to inform the matching decision.

In keeping with this explanation, the bias to respond ‘same’ was only apparent when participants viewed static faces in our study. When responding to a voice followed by a dynamic face, participants exhibited a bias to respond ‘different’. While voices are relatively weak signals to identity, they provide reliable speech information, and thus share a role with dynamic articulating faces. The additional information provided by dynamic compared to static faces influences the direction of the bias: It enables participants to use speech information to inform their decision, increasing the specificity and utility of the voice representation. This perhaps makes participants believe that they now have sufficient information to inform a ‘mismatch’ decision.

We suggest that some part of what is observed here could be partially driven by linguistic cues, despite participants being told that the linguistic content of the sentences does not matter. In our study, each sentence used in the experiment was unique and thus the linguistic content was never matched across the face and voice stimuli within a trial. Speech is readily comprehensible from audio clips, yet – for hearing participants – it is minimally intelligible from silent videos. Consequently, when hearing a voice first, the participant perceives a highly intelligible sentence that can in principle be compared with the movements on the lips in the following video. When the face produces a different sentence, and in the absence of the ability to integrate (non-speech) identity cues across modalities, the participant may be more inclined to give a ‘different identity’ response. This bias would, in contrast, be less pronounced for face-first trials where visual speech cues are less reliable and hence less constraining, and altogether absent for static images where speech cues were not present. Although participants were made aware that sentences did not repeat, and thus matching the linguistic content was not an appropriate strategy, they may well have allowed this to influence their decisions. Furthermore, we also do not claim that participants were using speech cues to build accurate perceptual models of a talker’s identity: Our analyses of overall accuracy show clearly that participants showed no above-chance accuracy, whether the mouth dynamics were visible or not. Whether linguistic or speech movement related cues can indeed modulate participants’ response biases could be empirically addressed in future work: For example, participants could be instructed to either pay attention to the linguistic speech content of the voice stimuli or could be asked to ignore it (as was the case in this experiment). If each sentence used in the study is unique, such that the linguistic content is different between the voice and dynamic face stimulus, response biases to say ‘different’ should be exaggerated for the condition when participants are encouraged to process the speech. Similarly, if a condition was introduced in which the sentences used for the face and voice stimuli are linguistically the same, then response biases to say ‘same’ should be exaggerated in the condition in which participants are encouraged to process speech.

## Conclusion

In sum, our data suggest that accurate face-voice matching of unfamiliar identities may not be possible at all times. However, this does not mean that face-voice matching is impossible under all circumstances – several reports of above-chance performance for dynamic face-voice matching exist in the literature (e.g. Kamachi et al., 2003; Lachs & Pisoni, 2004; Smith et al., 2016a, 2016b). We would argue that our results suggest that this ability is likely to be weak and not generalisable across all identities. Our results also indicate that even though speech cues are shared across both modalities, they are not used effectively to inform face-voice matching decisions. Nevertheless, we present evidence that face and voice processing do interact: Intriguingly, our results suggest that despite chance-level performance, participant responses are far from random. We reveal significant differences in how people *attempt* face-voice matching across different conditions through an analysis of response bias. While people might infer information about one modality from the other, the information is not necessarily reliable or accurate when the face and voice are presented in isolation.
